# Investigating adolescent psychological wellbeing in an educational context using PISA 2018 Canadian data

**DOI:** 10.3389/fpsyg.2024.1416631

**Published:** 2024-08-09

**Authors:** Yan Liu, Natasha S. Maltais, Marina Milner-Bolotin, Svetlana Chachashvili-Bolotin

**Affiliations:** ^1^Department of Psychology, Carleton University, Ottawa, ON, Canada; ^2^Department of Curriculum and Pedagogy, The University of British Columbia, Vancouver, BC, Canada; ^3^Institute for Immigration and Social Integration, Ruppin Academic Center, Hadera, Israel

**Keywords:** adolescents, psychological wellbeing, academic achievement, PISA 2018, immigrants, mixed effects model

## Abstract

**Introduction:**

Adolescent psychological wellbeing has been identified as an important public health priority and one of the major challenges facing young people. However, few studies have examined the wellbeing of Canadian adolescents nationwide in the past decade, and even fewer have focused specifically on immigrant adolescents. This study aims to investigate Canadian adolescent psychological wellbeing (PWB) via nationally representative data from the Programme for International Student Assessment (PISA) 2018. We explored what social and educational factors were critical in predicting Canadian adolescents' PWB, how adolescents from immigrant families differed from their non-immigrant peers in their wellbeing, and how adolescents' PWB was related to their academic performance.

**Methods:**

A total of 22,651 Grade 8 Canadian students who participated PISA 2018 were included in this study (mean age of 15; 49.9% female; 26% immigrant students). Mixed effects modeling was adopted for data analysis.

**Results:**

Our results showed that various social and educational factors were associated with adolescent PWB, but these relationships varied depending on which aspect of PWB was examined. Immigrant adolescents were shown to have higher levels of PWB when student attitudes toward immigrants were more positive. Additionally, most aspects of PWB were important for achievement performance.

**Discussion:**

It is important to develop healthy and supportive school and disciplinary climates that foster students' sense of belonging. To further support the PWB of immigrant adolescents, educators can implement activities and integrate learning materials on cultural diversity into curricula, encouraging students to develop positive attitudes toward immigrants. Our findings on the PWB of Canadian adolescents could provide valuable insights for other countries with diverse populations, particularly those with significant immigrant communities.

## Introduction

Adolescent psychological wellbeing was identified as an important public health priority in the World Health Organization's (WHO) 2014 report, *Health for the World's Adolescents* (World Health Organization (WHO), 2014). However, the literature on Canadian adolescent psychological wellbeing is scattered; very few studies have examined this issue nationwide in the past decade and even fewer studies have examined this issue in an educational context given that adolescents spend most of their time at school. Additionally, some researchers found that adolescents from immigrant families had numerous challenges when adapting to another culture, which affected their wellbeing (Hilario et al., 2015; Herati and Meyer, 2020). Canada has a large proportion of immigrants, about a quarter of the population (Statistics Canada, 2022). Given that the Canadian population is becoming more diverse, there is an urgent need to update the research on the status of adolescent psychological wellbeing nationwide in Canada.

The present study aims to investigate what social and educational factors are crucial in predicting Canadian adolescent psychological wellbeing as well as how adolescents from immigrant families differ from their non-immigrant peers in their wellbeing. Additionally, psychological wellbeing has been shown to have both positive and negative influences on adolescent achievement performance (Buhs and Ladd, 2001; Ponzo, 2013). Given the importance of schooling in adolescent life, we also examined how adolescent psychological wellbeing could be associated with their academic performance.

A high level of psychological wellbeing can engage students in learning, lead to high academic achievement, and prepare them for future careers (Noble and McGrath, 2011; Gutman and Vorhaus, 2012; Cadime et al., 2016). Adolescent wellbeing has been a global concern, but most international wellbeing surveys have primarily focused on adults (Pople et al., 2015). Additionally, the conceptualization of psychological wellbeing for adolescents varies depending on the theoretical approach adopted (Clarke, 2020). For example, Diener (1984) introduced a model of subjective wellbeing, known as *Hedonic Wellbeing*, which included positive affect (positive emotions), negative affect (negative emotions), and cognitive evaluations (e.g., life satisfaction). Ryff (1989) and Ryff and Singer (2008) proposed *Eudaimonic Wellbeing*, which included multiple positive psychological functions, including self-acceptance, personal growth, purpose in life, positive relations with others, environmental mastery, and autonomy (e.g., ability to resist social pressures to think and act in a positive way). *Hedonic* and *Eudaimonic Wellbeing* have been considered as complementary psychological functions (Huta, 2015).

Although there are various definitions, researchers have reached a consensus that psychological wellbeing is a complex and multidimensional construct encompassing emotional, social, and cognitive functioning, not just overall life satisfaction and happiness (Seligman and Csikszentmihalyi, 2000; Keyes, 2005). The recent definitions for adolescents have also taken school contexts into account (Holsted, 2015). The Organization for Economic Co-operation and Development (OECD, 2017, 2019a) specified three aspects of psychological wellbeing, which included life satisfaction, affect (positive and negative affect), and subjective perceptions (e.g., self-efficacy and fear of failure), and considered that psychological wellbeing was interrelated to other aspects of wellbeing, such as cognitive, social, and physical functioning and capabilities that students need to live a happy and fulfilling life. OECD's theoretical framework incorporates both Hedonic and Eudaimonic Wellbeing. Of note, we used PWB as an abbreviation for psychological wellbeing throughout the rest of this paper.

In this study, we adopted the theoretical view from OECD (2017, 2019a) and focused on adolescents' PWB, especially positive affect, self-efficacy, and fear of failure. Positive and negative emotions are considered as affective aspects of wellbeing in Diener's Hedonic Wellbeing model. Fredrickson (1998)'s “broaden-and-build” theory of *positive affect* relates to positive emotions, such as joy, which contribute to individuals' success. On the contrary, *negative affect* involves the experience of negative emotions, e.g., anger, guilt, disgust, which may lead to high levels of anxiety and stress and have a negative influence on students' academic performance (Laptook et al., 2008; Valiente et al., 2012; Russell et al., 2021).

*Self-efficacy*, originally introduced by Bandura (1977), has been widely used in psychology to study individuals' belief in their capacity/ability to execute a plan of action to achieve a particular goal. Low self-efficacy has been shown to lead to greater anxiety, depressive symptoms, and avoidant behaviors, which results in less successful coping with stress (Bandura, 1988; Bandura et al., 1999; Muris, 2002). Students with higher levels of self-efficacy tend to have better academic performance, better coping with stress, higher overall life satisfaction, and more commitment to remaining in school (Schunk and Ertmer, 2000; Pintrich, 2003; Ikiz, 2013).

*Fear of failure* is associated with *autonomy* in Eudaimonic Wellbeing. Autonomy refers to being able to resist social pressures to think and act positively, whereas fear of failure characterizes individuals' tendency to avoid making mistakes or avoid to take risks due to the perceived shame and uncertainty about the future (Windle, 2011; Hu et al., 2015). Adolescents developed fear of failure for a variety of reasons, such as extrinsic motivation, perfectionism, and pressure to succeed from parents, teachers, peers, and or self (Conroy, 2003; Alabduljabbar et al., 2022). Fear of failure has been shown to relate to shame, depression, anxiety, panic attacks, and low self-esteem (Sagar and Stoeber, 2009; Gustafsson et al., 2017). It was also found to be negatively related to achievement performance, such as reading and mathematics (Wang et al., 2019; Borgonovi and Han, 2021; Jeynes, 2022).

Numerous studies have investigated the impact of various social and educational elements on both life satisfaction and academic achievement. Several factors have been studied frequently, including sense of belonging, exposure to bullying, and growth mindsets. For example, adolescents were more likely to report higher levels of life satisfaction and better school performance if they had a *sense of belonging* in their school community and perceived the *school environment* positively (Liu et al., 2006; Aldridge et al., 2016; Gempp and González-Carrasco, 2021; Marquez, 2024). *Exposure to bullying* has been found to reduce adolescent life satisfaction and increase depression, anxiety, and suicidality (Diener, 2000; Schoeler et al., 2018; Pabian et al., 2022) as well as impede classroom learning and academic performance (Luiselli et al., 2005; Ponzo, 2013). Furthermore, several studies have revealed that, compared to students with fixed mindsets, those embracing growth mindsets are more inclined to establish mastery goals that emphasize the learning process and to have heightened self-efficacy, which results in improved academic performance (Costa and Faria, 2018; Sisk et al., 2018; Dweck and Yeager, 2019; Burnette et al., 2020; Lou et al., 2022).

Additional studies have shown that demographic characteristics, especially gender, economic, social, and cultural status (ESCS), and school type, could greatly affect adolescent PWB. McGregor and Elliot (2005) and Alkhazaleh and Mahasneh (2016) showed that women had higher levels of fear of failure than men. Other researchers found that women had lower self-efficacy and responded less positively to competitive environments (Niederle and Vesterlund, 2010; Huang, 2013; Goldman and Penner, 2016). ESCS is associated with student wellbeing and achievement. For example, ESCS was positively related to adolescent wellbeing (Korhonen et al., 2016). Socioeconomically privileged students normally had higher achievement than their underprivileged counterparts (Scerbina et al., 2019). There have been mixed findings about the role of school type (public vs. private). Some studies showed that students in private schools performed better than their public school peers (Martin-Chang et al., 2011; Jeynes, 2022), while other studies did not find any differences (Elder and Jepsen, 2014; Sakellariou, 2017). Hence, we included gender, ESCS, and school type in this study as control variables.

Most existing studies investigated how positive affect, self-efficacy, and fear of failure were related to adolescent academic performance. Additionally, most existing wellbeing studies focused on life satisfaction, depression, and anxiety. Fewer studies, however, have examined how social and educational factors affect positive affect, self-efficacy, and fear of failure for adolescents. One objective of this study is to provide insight into this issue, and our findings may inform educators about what interventions can be developed to help improve adolescent wellbeing and academic performance.

Research has found that children in immigrant families are more likely to be educationally disadvantaged as they may face additional obstacles such as language barriers, discrimination, and limited access to resources and opportunities (Andon et al., 2014; Bruckauf, 2016; Chachashvili-Bolotin et al., 2019; Chachashvili-Bolotin and Kreiner, 2022). Some researchers showed that young immigrant students were at a higher risk of failure and school dropout compared to their native peers (Andon et al., 2014; Schnell and Azzolini, 2015; Motti-Stefanidi, 2018). Using Programme for International Student Assessment (PISA) 2018 data, Rodríguez et al. (2020) found that native Spanish students had significantly higher positive affect and self-efficacy as well as higher levels of mathematics and science skills. They also found that first-generation immigrants had less desirable wellbeing levels and achievement performance than their second-generation counterparts. Additionally, immigrant-related discrimination has been shown to affect children and adolescents' academic performance and wellbeing (e.g., Guerra et al., 2019; Fu et al., 2021). However, little research has explored the association between adolescents' attitudes toward immigrants and immigrant adolescent wellbeing. This study investigated how adolescents' attitudes toward immigrants moderated the wellbeing differences between immigrant and non-immigrant adolescents.

Inconsistent findings have merged regarding the wellbeing status of immigrant youth in Canada. For instance, using provincial survey data, Smith et al. (2011) found no statistical differences in the mental health status between immigrant youth and youth born in Canada. On the contrary, with different provincial survey data, Hamilton et al. (2009) found that immigrant youth report higher rates of emotional distress compared to their Canadian-born peers. Using scoping reviews, Hilario et al. (2015) and Petrovskaya and Salami (2021) examined young immigrant youth's mental health in Canada and found that their mental health was dependent on their social and economic factors. However, most prior studies either used local data, which was not representative of the national population or used old national databases which have not been updated in the past decade. For example, the New Canadian Children and Youth Study (NCCYS) and the National Longitudinal Survey of Children and Youth (NLSCY) have been frequently used for studying youth wellbeing in Canada, but both databases have not been updated since 2010. Hence, there is a need to examine this issue nationwide with more updated data, which may inform what support or intervention programs are needed for young immigrants. To fill in this gap, the present study used the latest nationwide data from an international database, PISA 2018.

## Research questions

**Figure 1:**
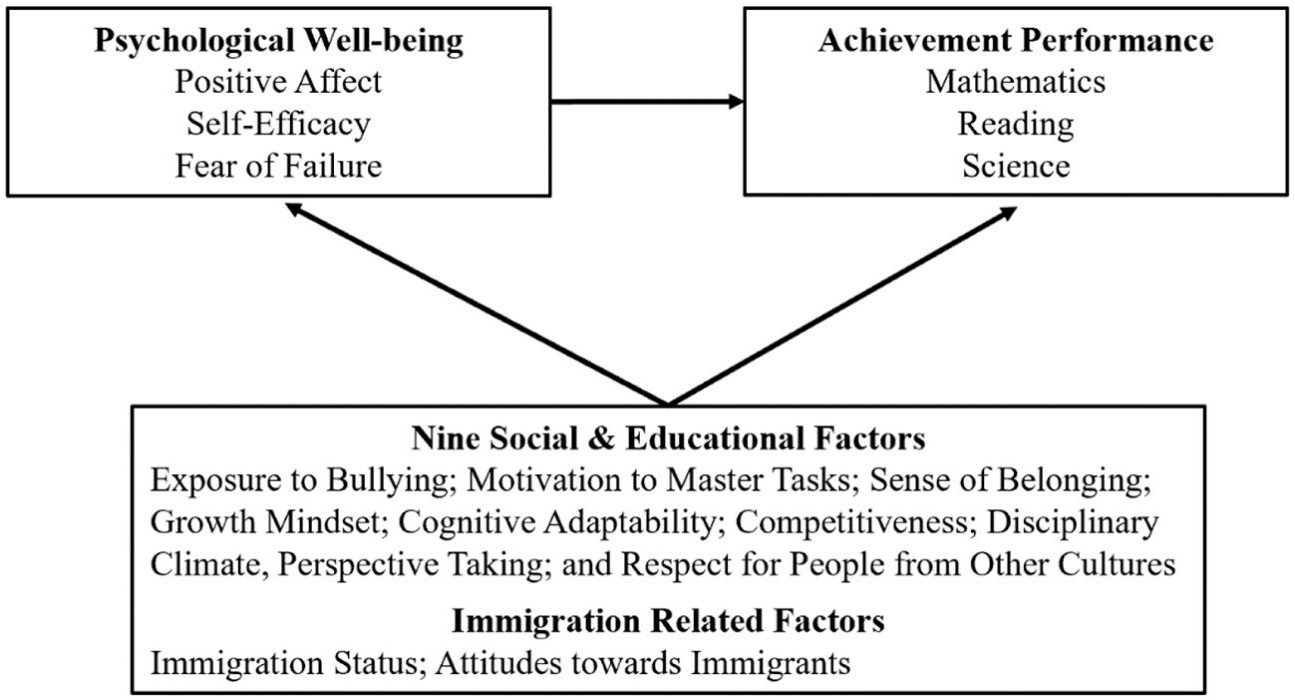
Elaboration of research questions with outcome variables and predictors.

This study aims to explore the critical social and educational factors in predicting the PWB of Canadian adolescents as well as to examine how adolescents' PWB influences their academic performance. Three aspects of PWB were studied, i.e., positive affect, self-efficacy, and fear of failure; and three achievement outcomes were investigated, i.e., mathematics, reading, and science. We included nine social and educational factors and examined how adolescents from immigrant families differed from their peers in wellbeing. Figure 1 elaborates on our research questions with all outcome variables and predictors. Additionally, to statistically control for differences in students' gender, family, and school background, we also included five control variables: gender, language at home, ESCS, grade repetition, and school type (public vs. private).

RQ1. How do various social and educational factors predict adolescent psychological wellbeing (positive feelings, self-efficacy, and fear of failure)?
RQ2. How do adolescents from immigrant families differ from their peers on their psychological wellbeing?
RQ3. How do student attitudes toward immigrants moderate the difference between immigrant adolescents and their non-immigrant peers on psychological wellbeing?
RQ4. How is adolescent psychological wellbeing associated with their achievement performance (mathematics, reading, and science)?

## Methods

### Data source

The data set used in this study was retrieved from the PISA 2018 database, https://www.oecd.org/pisa/data/2018database/. PISA is a triennial, international program hosted by OECD for measuring 15-year-old students' mathematics, reading, and science knowledge and skills to meet real-life challenges. The PISA 2018 also included questionnaires to measure students' wellbeing, demographic, family, and school-related factors. Seventy-nine countries and regions participated in PISA 2018.

Our study used Canadian data. A total of 22,651 Grade 8 students with an average age of 15 years old (49.9% female) participated in PISA 2018, and excluding those with missing data, 26% of the students identified themselves as having an immigrant background.^1^ The students were sampled from 821 schools across the 10 provinces in Canada. A two-stage stratified sampling design was adopted in PISA data collection to ensure the representativeness of the sample, with schools being sampled using probability proportional to their size, and students being sampled with equal probability within schools (OECD, 2019a).

### Measures and variables

PWB variables and other predictors were selected from the student questionnaire, and students' cognitive scores (mathematics, reading, and science) were obtained from student cognitive assessments. Detailed information on all the variables and measures can be found in Supplementary material.

#### Outcome variables

OECD (2017, 2019a) specified three aspects of PWB, (i) students' life satisfaction and meaning in life, (ii) students' feeling/affect (positive and negative affect), and (iii) subjective perceptions (e.g., self-efficacy and fear of failure). However, given that Canadian students did not respond to life satisfaction related questions, we only included the other two aspects. Additionally, we did not include negative affect for two reasons: positive affect is easy to incorporate into interventions and the negative affect scale had a low internal consistency across PISA-participating countries in the PISA 2018 (OECD, 2019a).

In the PISA 2018 data, OECD researchers provided indices, which are equivalent to scaled total scores of all items, for all three PWB variables. The *positive affect index* was derived from three 4-point Likert scale items (never, rarely, sometimes, and always), which asked how frequently students felt happy, joyful, and cheerful. The *self-efficacy index* was derived from five 4-point Likert scale items (strongly disagree, disagree, agree, and strongly agree) that measured students' belief in their ability to succeed and handle challenges. Positive values suggest higher positive feelings and self-efficacy than the average student across the participating OECD countries. The *fear of failure index* was computed from three 4-point Likert scale items (“strongly disagree” to “strongly agree”) that measured students' lack of confidence in their abilities following failure. Positive values mean that students expressed greater fear of failure and less confidence in their abilities than did the average student across OECD countries.

The cognitive assessment was used to assess students' mathematics, reading, and science competencies. Due to the time constraints, each student only responded to part of each test. For each test, 10 plausible values, ranging from 1 to 1,000 with a mean of 500, were estimated for each student to approximate their cognitive abilities by PISA researchers, using item response theory scaling methods (OECD, 2017).

#### Predictors

Nine educational and social factors were included as the primary predictors to address RQ1–RQ3. Like PWB variables, the indices provided by PISA researchers were used in this study except for *growth mindset* which is an individual variable. In general, the positive values indicate that students scored higher compared to the average student across OECD countries.

Four educational factors were included. The *competitive index* was derived from three 4-point Likert scale items (“strongly disagree” to “strongly agree”), which measured students' achievement motive and enjoyment in a competitive environment. The *disciplinary climate index* was based on five 4-point Likert scale items (every lesson, most lessons, some lessons, never, or hardly ever), asking how often students experienced positive or negative climates in the class. The *motivation to master tasks index* (Motivation) measured students' motivation to work hard, derived from three 4-point Likert scale items (“strongly disagree” to “strongly agree”). The *cognitive adaptability index* measured students' adaptability or flexibility in dealing with challenging or difficult situations, which include intercultural situations. It was computed from five 5-point Likert items (very much like me, mostly like me, somewhat like me, not much like me, not at all like me).

Five social factors were included. The *exposure to bullying index* was derived from three 4-point Likert scale items (“never or almost never” to “once a week or more”), which asked students about the frequency of their experiences with bullying at school. The *sense of belonging index* was computed from six 4-point Likert scale items (“strongly disagree” to “strongly agree”), which measured student perceptions about their belonging at school and relationships with peers. The *perspective taking index* assessed how far a person takes the perspective of other people. Using PISA research framework, perspective taking is seen as an important precondition for successful intercultural relationships. It was derived from four 5-point Likert scale items (“very much like me” to “not at all like me”). The *respect for people from other cultures index* (Respect) was defined as having positive regard and esteem for those people because they were perceived to have an intrinsic importance, worth, or value that stemmed from their inherent dignity as human beings. This index may be related to students' attitudes toward to immigrants, which may indirectly affect immigrant students' PWB. It was based on four 5-point Likert scale items (“very much like me” to “not at all like me”).

The *growth mindset* variable was obtained from one single 4-point Likert item (“strongly disagree” to “strongly agree), which asked students to respond to “Your intelligence is something about you that you can't change very much.” A binary categorical variable used in the present study was also provided by PISA researchers to describe whether students had a growth mindset or not.

#### Immigrant related variables

To address RQ2 and RQ3, we included attitudes toward immigrants and immigration status. The *attitudes toward immigrants' index* (AttdImg) measured students' opinions on the opportunities and rights of immigrants, derived from four 4-point Likert scale items (“strongly disagree” to “strongly agree”). The higher values suggest more positive attitudes. *Immigration status* is a categorical variable that indicates the immigration status of the student, including native students, first-generation immigrant students (those students and their parents born outside of Canada), and second-generation immigrant students (those born in Canada, but with at least one parent born in another country). Using native students as the reference, we created two dummy coded variables for immigration status, *Img1Gen* (first generation immigrants) and *Img2Gen* (second generation immigrants).

#### Control variables

Five variables were used in this study to control for differences in students' gender, family, and school background, which included gender, language at home, ESCS, grade repetition, and school type. Gender is named *Male* in our results (0 = girls; 1 = boys). *Language at home* (Language) is a categorical variable based on the language spoken at home by the student, including English, French, and others. Two dummy variables were created using English as the reference group. *ESCS* is a continuous composite score based on 3 indexes that measured parental occupation, parental education, and home possessions. Home possessions were used as a proxy for family wealth. Positive ESCS values mean that the student had a higher economic, social, and cultural status than the average student across OECD countries.

The *grade repetition index* is a binary categorical variable (No vs. Yes), derived from three 3-point Likert scale items (“no/never,” “yes, once,” and “yes, twice or more”). It measured the repetition of the students at different grades in school. *School type* (Public school) originally had three categories, private independent, private government-dependent, and public. We recorded this variable to be a dummy variable (private = 0, public = 1) due to the small number of private government-dependent schools (3.6%). It was the only variable collected from the school level.

### Data analysis

Based on the missing at random assumption, missing values in the data set were inputted using random forests algorithm, implemented by R package *missRanger* (Mayer, 2023) in R version 4.2.3 (R Core Team, 2023). The proportions of missing values ranged from 0.01% to 16.92%. The three achievement outcomes had no missingness. This imputation approach has shown similar performance as multiple imputation (Waljee et al., 2013).

Sampling weights were used to account for differences in the probabilities of students being selected in the sample and to ensure that the sample represented the target population. Following recommendations from Lorah (2022), we only used school weights provided by PISA data in the data analysis. Although we included student weights as a variable in Mplus syntax, this variable consisted of values of 1 that were used to make the code work.

Linear mixed effects modeling, also known as multilevel modeling, was performed to address our research questions. M*plus* 8 (Muthén and Muthén, 1998-2023) was used for the data analysis. To account for the cluster effect resulting from the sampling design in PISA data collection, two-level models with random intercepts were employed. We added variables sequentially, including the null model, 2^nd^ model (adding control variables only), and 3^rd^ model (adding the primary predictors). Detailed information about the results of these models can be found in Supplementary material. We only provided the results obtained from the final model in this paper. To handle 10 plausible values for each achievement outcome, we followed Aparicio et al. (2021)'s recommendations, conducting the analysis for each of the plausible values, and then integrating the ten sets of results, which is similar to how one handles multiple imputed data sets.

## Results

Descriptive statistics of all the variables are presented in Table 1. All the continuous study variables showed approximately normal distribution with skewness ranging from −1.31 to 0.96 and kurtosis ranging from −0.70 to 2.29. English speakers were the dominant group (64.52%), French speakers (17.03%) were the next, and all other language speakers were combined into one group (13.53%). With about 7% missing values on the immigration status, 13.56% of students identified themselves as first-generation immigrants and 10.83% as second-generation immigrants. Only 5.09% of students indicated that they repeated grade. More than half (59.62%) of students believed that they had a growth mindset. Most students attended public schools (90.91%).

**Table 1:** Descriptive statistics for original sample data.

	***n***	**Mean**	***SD***	**Median**	**Skew**	**Kurtosis**	**Missing %**
**Outcome variables**							
Positive affect	19,989	−0.06	0.96	0.10	−0.28	−0.70	11.76
Student self-efficacy	20,090	0.13	0.95	−0.06	0.46	0.37	11.31
Fear of failure	20,169	0.27	0.98	0.46	−0.13	−0.60	10.97
Mathematics scores	22,653	512.02	92.29	513.48	−0.08	−0.07	-
Reading scores	22,653	520.09	100.30	524.08	−0.20	−0.17	-
Science scores	22,653	518.00	95.72	520.37	−0.13	−0.15	-
**Continuous predictors**							
ESCS	21,490	0.42	0.79	0.53	−0.47	0.85	5.13
Disciplinary climate	21,353	−0.09	1.01	−0.04	−0.13	0.16	5.74
Being bullied	18,820	0.14	0.96	0.15	0.88	0.31	16.92
Motivation	19,999	0.12	0.92	−0.10	−0.03	−0.23	11.72
Sense of belonging	20,091	−0.18	0.91	−0.32	0.96	2.29	11.31
Cognitive adaptability	19,762	0.20	0.94	0.21	0.28	0.06	12.76
Competitiveness	20,213	0.16	0.98	0.20	0	−0.12	10.77
Perspective taking	19,782	0.14	0.92	0.14	0.06	0.06	12.67
Respect	19,577	0.30	0.81	0.93	−1.31	1.10	13.58
Attitudes toward immigrants	19,332	0.46	0.90	0.60	−0.59	−0.36	14.66
	*n*	%	Missing %			
**Categorical predictors**							
Gender (F = 0, M = 1)	22,651	50.08	0.01			
Language at Home			4.91			
English	14,616	64.52				
French	3,858	17.03				
Other	3,066	13.53				
Immigration			7.23			
First generation	3,072	13.56				
Second generation	2,453	10.83				
Native	15,491	68.38				
Grade repetition (No = 0, Yes = 1)	20,727	5.09	8.50			
Growth mindset (No = 0, Yes = 1)	20,250	59.62	10.61			
School type (private = 0, public = 1)	22,549	90.91	0.46			

ESCS, Index of economic, social, and cultural status; Motivation, Motivation to master tasks; Respect, Respect for people from other cultures. The descriptive statistics are based on the original sample before imputation.

The rest of this section is organized by four research questions. We conducted mixed effects models for three PWB outcome variables and three achievement outcome variables. Intra-class correlations (ICCs) were found to be relatively large for all achievement outcomes, ranging from 0.161 to 0.193, but ICCs were small for wellbeing outcomes, ranging from 0.013 to 0.025, which may indicate that there was not much school variation in wellbeing outcomes.

### RQ1: relationships of social and educational factors and PWB

Table 2 presents the results for RQ1. The bottom of Table 2 reports R^2^ at both within- and between-levels. The R^2^ values were relatively large at the within-level (0.222 for positive affect; 0.375 for self-efficacy; 0.156 for fear of failure), but only positive affect had a relatively large R^2^ value (0.225) at the between-level.

**Table 2:** The relationships of three psychological wellbeing outcomes and social and educational factors via linear mixed effects models.

	**Positive affect**			**Self-efficacy**			**Fear of failure**		
	**Estimate**	***SE***	***t*** **-test**	**Estimate**	***SE***	***t*** **-test**	**Estimate**	***SE***	***t-*** **test**
**Fixed effects**									
***Control variables***									
Male	−0.005	0.021	−0.264	0.093	0.017	5.366^***^	−0.471	0.025	−19.057^***^
Language (French)^a^	0.168	0.025	6.630^***^	0.079	0.026	3.079^**^	−0.111	0.027	−4.183^***^
Language (Other)^a^	0.063	0.027	2.336^*^	0.082	0.024	3.470^**^	−0.035	0.028	−1.251
ESCS	0.027	0.013	2.029^*^	0.034	0.010	3.313^**^	0.066	0.013	5.161^***^
Grade Repetition	0.033	0.044	0.748	0.015	0.053	0.282	−0.041	0.052	−0.796
Public school	0.107	0.034	3.157^**^	−0.009	0.036	−0.247	0.042	0.034	1.223
***Primary predictors***									
Sense of belonging	0.327	0.012	26.696^***^	0.160	0.010	15.724^***^	−0.160	0.014	−11.461^***^
Exposure to bullying	−0.094	0.009	−9.866^***^	−0.013	0.009	−1.406	0.102	0.013	8.156^***^
Competitiveness	0.041	0.011	3.695^***^	0.098	0.011	9.221^***^	0.104	0.011	9.612^***^
Disciplinary climate	0.020	0.010	1.994^*^	0.020	0.009	2.128^*^	0.009	0.012	0.742
Motivation	0.135	0.011	11.991^***^	0.311	0.012	26.448^***^	0.088	0.013	6.970^***^
Growth mindset	0.012	0.025	0.462	0.032	0.019	1.654	−0.191	0.020	−9.334^***^
Cognitive adaptability	0.081	0.013	6.326^***^	0.293	0.013	22.627^***^	−0.134	0.013	−10.423^***^
Perspective taking	−0.016	0.013	−1.252	−0.005	0.010	−0.451	0.071	0.013	5.598^***^
Respect	0.032	0.015	2.066^*^	−0.005	0.012	−0.418	0.116	0.014	8.066^***^
**Random effects**									
Residual variance	0.837			0.633			0.922		
School variance	0.005			0.005			0.004		
**R Square**									
Within-level	0.222			0.376			0.156		
Between-level	0.225			0.002			0.053		

ESCS, Index of economic, social, and cultural status; Motivation, Motivation to master tasks; Respect, Respect for people from other cultures; ^a^Reference category is English; ^*^p ≤ 0.05, ^**^p <0.01, ^***^p <0.001.

Using linear mixed effects models, we found that four out of nine social and educational factors were statistically significant for all PWB outcomes, including *sense of belonging, competitiveness, motivation to master tasks, and cognitive adaptability*. All these factors showed a positive relationship with all outcomes except that two predictors were negatively associated with fear of failure (*sense of belonging*: b = −0.16, *p* < 0.001; *cognitive adaptability*: b = −0.134, *p* < 0.001). The rest of the predictors played different roles in the three wellbeing outcomes.

*Exposure to bullying* had a negative relationship with positive affect (b = −0.094, *p* < 0.001) and a positive relationship with fear of failure (b = 0.102, *p* < 0.001. *Disciplinary climate* was only positively associated with positive affect (b = 0.02, *p* = 0.046) and self-efficacy (b = 0.02, *p* = 0.033). *Growth mindset* was negatively associated with fear of failure (b = −0.191, *p* < 0.001), but *perspective taking* was positively associated with fear of failure (b = 0.071, *p* < 0.001). *Respect for people from other cultures* was positively associated with both fear of failure (b = 0.116, *p* < 0.001) and positive affect (b = 0.031, *p* = 0.039).

Gender differences were found, with boys having higher self-efficacy (b = 0.093, *p* < 0.001) and lower fear of failure (b = −0.471, *p* < 0.001) than girls. Compared to English speakers, French speakers were found to have a higher positive affect (b = 0.168, *p* < 0.001) and self-efficacy (b = 0.079, *p* = 0.002), but lower fear of failure (b = −0.111, *p* < 0.001) on average. Other language speakers were found to have higher positive affect (b = 0.063, *p* = 0.02) and self-efficacy (b = 0.082, *p* = 0.001). ESCS was a significant control variable for all outcomes, while grade repetition was not statistically significant for all outcomes. School type was only related to positive affect, i.e., students in public schools were more likely to have more positive affect (b = 0.107, *p* = 0.002).

### RQ2: differences between immigrant students and their non-immigrant peers

By adding *immigrant status* to the model, the random effects and most R^2^ values did not change, except that R^2^ increased slightly for positive affect (between-level ΔR^2^ = 0.008). The tiny R^2^ change suggests that adding immigrant status did not explain much more variation in the wellbeing outcomes. The relationships of all variables included in the previous model remained the same. Table 3 shows that immigrant students did not statistically differ from native students in positive affect and self-efficacy. The second generation of immigrant students (*Img2Gen*) showed a higher level of fear of failure (b = 0.085, *p* = 0.011) than native students, but not the first generation (*Img1Gen*).

**Table 3:** Comparing immigrant and non-immigrant students on three wellbeing variables via linear mixed effects models.

	**Positive affect**			**Self–efficacy**			**Fear of failure**		
	**Estimate**	***SE***	***t-*** **test**	**Estimate**	***SE***	***t-*** **test**	**Estimate**	***SE***	***t*** **-test**
**Fixed effects**									
***Control variables***									
Male	−0.006	0.021	−0.288	0.092	0.017	5.305^***^	−0.473	0.025	−19.080^***^
Language (French) ^a^	0.169	0.025	6.664^***^	0.082	0.025	3.224^**^	−0.107	0.027	−4.006^***^
Language (Other) ^a^	0.052	0.035	1.493	0.056	0.028	1.979^*^	−0.045	0.031	−1.421
ESCS	0.027	0.013	2.040^*^	0.034	0.010	3.355^**^	0.067	0.013	5.238^***^
Grade repetition	0.032	0.044	0.732	0.014	0.053	0.265	−0.038	0.052	−0.733
Public school	0.109	0.034	3.209^**^	−0.004	0.035	−0.117	0.047	0.034	1.388
***Primary predictors***									
Sense of belonging	0.327	0.012	26.699^***^	0.160	0.010	15.707^***^	−0.160	0.014	−11.495^***^
Exposure to bullying	−0.094	0.009	−9.876^***^	−0.013	0.009	−1.414	0.102	0.013	8.147^***^
Competitiveness	0.041	0.011	3.660^***^	0.098	0.011	9.171^***^	0.104	0.011	9.550^***^
Disciplinary climate	0.019	0.010	1.980	0.019	0.009	2.037^*^	0.009	0.012	0.738
Motivation	0.135	0.011	11.991^***^	0.311	0.012	26.470^***^	0.088	0.013	6.950^***^
Growth mindset	0.011	0.026	0.426	0.029	0.019	1.539	−0.194	0.021	−9.433^***^
Cognitive adaptability	0.081	0.013	6.318^***^	0.293	0.013	22.648^***^	−0.133	0.013	−10.376^***^
Perspective taking	−0.016	0.013	−1.260	−0.005	0.010	−0.497	0.070	0.013	5.489^***^
Respect	0.031	0.015	2.057^*^	−0.005	0.012	−0.452	0.115	0.014	7.998^***^
Img1Gen	0.019	0.036	0.512	0.040	0.031	1.281	−0.002	0.028	−0.088
Img2Gen	0.016	0.029	0.572	0.040	0.022	1.788	0.085	0.033	2.552^*^
**Random effects**									
Residual variance	0.837			0.633			0.922		
School variance	0.005			0.005			0.004		
**R square**									
Within-level	0.222			0.376			0.157		
Between-level	0.233			0.001			0.075		

ESCS, Index of economic, social, and cultural status; Motivation, Motivation to master tasks; Respect, Respect for people from other cultures; Img1Gen, 1^st^ generation immigrants; Img2Gen, 2^nd^ generation immigrants; ^a^Reference category is English; ^*^p ≤ 0.05, ^**^p <0.01, ^***^p <0.001.

### RQ3: moderation effects of attitudes toward immigrants

After adding *attitudes toward immigrants* (AttdImg) and interactions, Img1Gen^*^AttdImg and Img2Gen^*^AttdImg, random effects at the within-level reduced slightly for self-efficacy and fear of failure, while R^2^ increased slightly for positive affect (between-level ΔR^2^ = 0.008), self-efficacy (between-level ΔR^2^ = 0.001), and fear of failure (between-level ΔR^2^ = 0.001; within-level ΔR^2^ = 0.022). The small R^2^ changes suggest that the effect size of the moderation effect was small.

Table 4 shows the moderation effect of attitudes toward immigrants was statistically significant for both positive affect and self-efficacy, but not for fear of failure. Student attitudes toward immigrants moderated the difference between the second generation of immigrant students and native students in positive affect (Img1Gen^*^AttdImg: b = 0.071, *p* = 0.035) and in self-efficacy (Img1Gen^*^AttdImg: b = 0.072, *p* = 0.019) as well as moderated the difference between the first generation and native students in self-efficacy (Img2Gen^*^AttdImg: b = 0.056, *p* = 0.016). For fear of failure, we only found the main effect of student attitudes toward immigrants (b = 0.061, *p* < 0.001). After adding the moderation effects, all variables remained the same relationships with the wellbeing outcomes except that other language speakers did not have any differences on all outcomes from English speakers anymore.

**Table 4:** The moderation effect of attitudes toward immigrants on three psychological wellbeing variables via linear mixed effects models.

	**Positive affect**			**Self–efficacy**			**Fear of failure**		
	**Estimate**	***SE***	***t*** **-test**	**Estimate**	***SE***	***t*** **-test**	**Estimate**	***SE***	***t*** **-test**
**Fixed effects**									
***Control variables***									
Male	−0.005	0.021	−0.230	0.095	0.017	5.533^***^	−0.462	0.025	−18.571^***^
Language (French) ^a^	0.171	0.025	6.772^***^	0.084	0.025	3.297^**^	−0.108	0.027	−4.030^***^
Language (Other) ^a^	0.051	0.035	1.458	0.055	0.028	1.930	−0.047	0.031	−1.508
ESCS	0.027	0.013	2.073^*^	0.034	0.010	3.421^**^	0.064	0.013	5.012^***^
Grade repetition	0.036	0.045	0.804	0.018	0.053	0.346	−0.032	0.052	−0.612
Public school	0.113	0.034	3.349^**^	0.001	0.035	0.015	0.049	0.033	1.461
***Primary predictors***									
Sense of belonging	0.327	0.012	26.586^***^	0.159	0.010	15.584^***^	−0.162	0.014	−11.808^***^
Exposure to bullying	−0.093	0.009	−9.856^***^	−0.012	0.009	−1.324	0.103	0.013	8.218^***^
Competitiveness	0.040	0.011	3.657^***^	0.097	0.011	9.188^***^	0.103	0.011	9.582^***^
Disciplinary climate	0.019	0.010	1.985^*^	0.019	0.009	2.026^*^	0.008	0.012	0.696
Motivation	0.133	0.011	11.734^***^	0.307	0.012	25.982^***^	0.081	0.013	6.360^***^
Growth mindset	0.011	0.025	0.440	0.029	0.019	1.533	−0.195	0.020	−9.567^***^
Cognitive adaptability	0.082	0.013	6.387^***^	0.294	0.013	22.813^***^	−0.130	0.013	−10.192^***^
Perspective taking	−0.017	0.013	−1.292	−0.006	0.010	−0.567	0.068	0.013	5.301^***^
Respect	0.026	0.017	1.527	−0.016	0.013	−1.253	0.086	0.015	5.888^***^
Img1Gen	−0.016	0.043	−0.363	0.003	0.038	0.091	−0.024	0.030	−0.776
Img2Gen	−0.004	0.035	−0.113	0.011	0.024	0.450	0.078	0.038	2.029^*^
AttdImg	−0.004	0.013	−0.310	0.004	0.013	0.341	0.061	0.014	4.265^***^
Img1Gen ^*^ AttdImg	0.071	0.034	2.109^*^	0.072	0.031	2.346^*^	0.022	0.027	0.840
Img2Gen ^*^ AttdImg	0.042	0.030	1.417	0.056	0.023	2.414^*^	−0.005	0.037	−0.121
**Random effects**									
Residual variance	0.837			0.632			0.918		
School variance	0.005			0.005			0.004		
**R Square**									
Within-Level	0.223			0.377			0.160		
Between-Level	0.246			0.001			0.076		

ESCS, Index of economic, social, and cultural status; Motivation, Motivation to master tasks; Respect, Respect for people from other cultures; Img1Gen, 1^st^ generation immigrants; Img2Gen, 2^nd^ generation immigrants; ^a^Reference category is English; ^*^p ≤ 0.05, ^**^p <0.01, ^***^p <0.001.

### RQ4: relationships of wellbeing and achievement performance

Our RQ4 aims to examine how adolescent PWB affects their achievement performance (mathematics, reading, and science) after controlling for all other predictors. Table 5 presents the results for RQ4. Similar to the results of wellbeing outcomes, R^2^ values for achievement outcomes were relatively large at the within-level (0.175 for mathematics; 0.221 for reading; 0.172 for science), but small at the between-level, ranging from 0.02 to 0.085.

**Table 5:** The relations of psychological wellbeing and achievement outcomes (mathematics, reading, and science) via linear mixed effects models.

	**Mathematics**			**Reading**			**Science**		
	**Estimate**	***SE***	***t-*** **test**	**Estimate**	***SE***	***t*** **-test**	**Estimate**	***SE***	***t*** **-test**
**Fixed effects**									
***Control variables***									
Male	18.548	2.353	7.883^***^	−7.133	1.957	−3.644^***^	13.477	2.751	4.900^***^
Language (French) ^a^	19.491	5.479	3.557^***^	3.921	4.306	0.911	6.329	4.701	1.346
Language (Other) ^a^	8.089	3.427	2.360^*^	−11.724	3.356	−3.494^***^	−9.896	3.940	−2.512^*^
ESCS	19.044	1.241	15.348^***^	16.456	1.224	13.443^***^	15.775	1.306	12.075^***^
Grade repetition	−50.316	5.382	−9.350^***^	−46.221	4.570	−10.113^***^	−44.754	4.991	−8.967^***^
Public school	−24.453	8.712	−2.807^**^	−17.106	9.279	−1.844	−11.420	10.205	−1.119
Sense of belonging	−3.957	1.639	−2.415^*^	−3.755	1.364	−2.753^**^	−3.246	1.491	−2.177^*^
Exposure to bullying	−6.991	1.180	−5.924^***^	−7.402	1.013	−7.310^***^	−6.369	1.372	−4.643^***^
Competitiveness	3.195	1.295	2.467^*^	2.330	1.028	2.268^*^	5.508	1.241	4.440^***^
Disciplinary climate	4.638	1.135	4.086^***^	5.488	1.087	5.050^***^	4.630	1.189	3.895^***^
Motivation	4.685	1.628	2.877^**^	6.441	1.157	5.568^***^	4.566	1.105	4.134^***^
Growth mindset	18.265	2.811	6.498^***^	24.227	2.199	11.016^***^	17.094	2.374	7.201^***^
Cognitive adaptability	0.800	1.314	0.609	0.363	1.227	0.295	2.261	1.360	1.662
Perspective taking	−1.591	1.833	−0.868	−1.339	1.140	−1.175	−0.905	1.316	−0.688
Respect	9.582	1.356	7.068^***^	15.618	1.347	11.596^***^	11.257	1.525	7.379^***^
Img1Gen	−14.377	4.195	−3.427^**^	−19.313	3.154	−6.123^***^	−16.695	3.763	−4.437^***^
Img2Gen	−9.719	4.382	−2.218^*^	−6.440	3.102	−2.076^*^	−9.305	3.542	−2.627^**^
AttdImg	9.447	1.431	6.604^***^	14.373	1.045	13.756^***^	13.076	1.525	8.575^***^
***Primary predictors***									
Positive affect	−6.168	1.422	−4.337^***^	−6.557	1.166	−5.622^***^	−6.904	1.441	−4.791^***^
Self-efficacy	4.639	1.778	2.609^**^	1.766	1.324	1.334	1.566	1.810	0.865
Fear of failure	2.637	0.947	2.785^**^	7.106	0.986	7.204^***^	5.917	1.149	5.149^***^
**Random effects**									
Residual variance	5,929.686			6,999.819			6,705.559		
School variance	891.884			845.044			914.334		
**R square**									
Within-level	0.175			0.221			0.172		
Between-level	0.085			0.046			0.020		

ESCS, Index of economic, social, and cultural status; Motivation, Motivation to master tasks; Respect, Respect for people from other cultures; Img1Gen, 1^st^ generation immigrants; Img2Gen, 2^nd^ generation immigrants; ^a^Reference category is English; ^*^p ≤ 0.05, ^**^p <0.01, ^***^p <0.001.

Positive affect and fear of failure were found to be statistically associated with all achievement outcomes. Contrary to the findings from previous research, *positive affect* was negatively related to all achievement outcomes (mathematics: b = −6.168, *p* < 0.001; reading: b = −6.557, *p* < 0.001; science: b = −6.904, *p* < 0.001), whereas *fear of failure* was positively related to all the outcomes (mathematics: b = 2.637, *p* = 0.005; reading: b = 7.106, *p* < 0.001; science: b = 5.917, *p* < 0.001). *Self-efficacy* was positively correlated with mathematics scores (b = 4.639, *p* < 0.001), but not correlated with reading and science scores.

Most control variables were found to be statistically significantly related to all three achievement outcomes, with a few exceptions. Both *cognitive adaptability* and *perspective taking* were not related to any achievement outcomes. *Language* (*French*) and *school type* were only related to mathematics scores but not related to other achievement outcomes. French speakers on average had higher mathematics scores than English speakers (b = 19.49, *p* < 0.001); students who attended public schools tended to have lower mathematics scores (b = −24.45, *p* = 0.005). It should be noted that all immigrant students had lower performance on all achievement outcomes than native students. Our results also showed that compared to boys, girls had higher performance in reading, but lower performance in mathematics and science.

## Discussion and conclusion

Adolescent wellbeing has been recognized as an important public health priority worldwide. However, few studies have examined Canadian adolescent wellbeing nationwide in the past decade and even fewer studies have examined this issue for immigrant adolescents. Using nationally representative data, our study investigated what social and educational factors predicted Canadian adolescents' PWB and how adolescents from immigrant families differed from their non-immigrant peers in their wellbeing. We also examined how adolescent wellbeing could affect their academic performance. Our results revealed that various social and educational factors were associated with adolescent wellbeing, but these relationships varied depending on which aspect of wellbeing was examined. Immigrant adolescents were shown to have higher levels of wellbeing when student attitudes toward immigrants were more positive. Additionally, all aspects of wellbeing were important to achievement performance except that self-efficacy was not statistically related to reading and science scores.

It is worth noting that ICCs were small for all wellbeing outcomes which indicates small variations at the school level for wellbeing outcomes. However, this finding does not mean that school factors are not important to adolescent wellbeing. It only indicates that adolescent wellbeing does not vary much across different schools, which may be due to support and services being evenly distributed across schools in Canada.

One interesting finding in our study was that higher competitiveness was associated with both higher wellbeing and achievement outcomes. Educators have a longstanding debate about whether competitions are good for student learning. Some researchers supported the use of competitions in learning and showed some positive evidence with empirical data, such as increased motivation, self-esteem, life satisfaction, and persistence when taking on challenges (Lawrence, 2004; Fasli and Michalakopoulos, 2005; Koyama and Fujiwara, 2023), whereas others suggested that it increased student anxiety, depression, and fear of failure, and decreased intrinsic motivation (Shindler, 2009; Chan and Cheung, 2022). Given that it is impossible to avoid competition throughout different life stages, it is better to prepare students for this. We believe that creating a *healthy* competitive environment is essential for adolescent wellbeing and academic performance. Educators can aim to develop students' intrinsic motivations (their interests in the subject), design problem-based learning, and emphasize both collaborative learning and competition.

Our findings suggest more research is needed to investigate the role of two important variables, *cognitive adaptability*, and *growth mindset*, given that the existing research is limited, and inconsistent results have been found in the literature. Our findings revealed that *cognitive adaptability* was important for all wellbeing outcomes, but not for achievement outcomes. Cognitive adaptability has been shown to be negatively associated with perceived stress, depression, and other psychological symptoms, but positively associated with problem-solving ability and academic performance (Martin et al., 2011; Marshall and Brockman, 2016; Stockinger et al., 2021). Our findings were consistent with the prior research on PWB, but not for achievement outcomes. It is possible that adaptability influences achievement performance through other variables (i.e., mediators). Feraco et al. (2022) suggested that cognitive adaptability indirectly influenced achievement performance via engagement and self-regulated learning. More research is needed to examine what role cognitive adaptability plays in achievement performance for adolescents.

Our results also showed that g*rowth mindset* was positively related to all achievement outcomes, but not related to wellbeing outcomes except for fear of failure. It is widely known that individuals with a growth mindset can have higher motivation and in turn, can improve their achievement performance. Lately, researchers have started to examine how growth mindset affects wellbeing, mostly focusing on stress, anxiety, and depression (Schroder et al., 2017; Schleider and Weisz, 2018; Dweck and Yeager, 2019). Very few studies have investigated how a growth mindset is related to positive affect and self-efficacy. We found one study in the literature showing a positive relationship between growth mindset and self-efficacy (Orvidas et al., 2018), which differs from our findings. Hence, more research is encouraged given that educators can incorporate cognitive adaptability and growth mindset into wellbeing interventions.

*Student attitudes toward immigrants* were found to moderate the differences in wellbeing between immigrant and native students for positive affect and self-efficacy (see Table 4). Our results showed that when students' attitudes toward immigrants became more positive, immigrant students had a higher positive affect and self-efficacy in general. However, without adding this moderation effect, we would not find any differences between immigrant and native students in positive affect and self-efficacy. Hence, we suggest researchers consider moderators when comparing immigrant students to native students in wellbeing studies.

Our findings also indicated distinctions between first and second-generation immigrant students in terms of positive affect and fear of failure. The first generation showed a notable increase in positive affect when the general attitudes of students toward immigrants held more positive, whereas this was not observed among the second generation. On the other hand, the second generation exhibited a greater fear of failure compared to native students, but there was no disparity in fear of failure between the first generation and native students. These results suggest that it could be more meaningful to differentiate between generations in wellbeing research, as their experiences appear to vary.

In addition, our findings revealed that the relationship between wellbeing and achievement may not always be positive. Many prior studies have provided empirical evidence to show that a high level of PWB can engage students in learning and lead them to high academic achievement. However, our study revealed contradictory findings: the higher the positive affect, the lower the achievement outcomes became; the higher the fear of failure, the higher the achievement outcomes became. Self-efficacy was only associated with mathematics scores. A few researchers have pointed out that exhaustive engagement may be positively related to short-term academic performance, but it may lead to burnout and stress in the long term, which can decrease academic performance (Walburg, 2014; Wang et al., 2019). This may be one potential explanation for our findings. On top of this, some researchers have also argued that wellbeing and achievement had a bidirectional relationship, which encourages longitudinal studies for further investigations (e.g., Bortes et al., 2021).

While our study employed rigorous methodology, it has some limitations. First, we did not include some other important factors, such as parent support, as Canadian students did not respond to some survey questions. We encourage researchers to include variables related to parent support in their studies, as it has been shown to be a crucial factor in adolescent well-being. Second, our study only examined a limited number of wellbeing variables. Canadian students did not respond to questions related to life satisfaction and meaning in life which limited how we measured PWB in this study. Additionally, the PISA 2018 survey had a separate questionnaire that included both physical and PWB, but Canadian students did not respond to this questionnaire. Researchers are encouraged to examine various aspects of wellbeing using other national data.

In conclusion, it is important to develop healthy and supportive school and disciplinary climates that foster students' sense of belonging. Based on our findings, educators can develop intervention programs to improve PWB by encouraging adolescents' growth mindset, motivation to master tasks, and cognitive adaptability. These programs could also promote a sense of belonging and create a healthy competitive environment within schools. To further support the wellbeing of immigrant adolescents, educators can implement activities and integrate learning materials on cultural diversity into curricula, encouraging students to develop positive attitudes toward immigrants. It is also important for educators to guide adolescents to take care of their needs for maintaining PWB and to maximize their potential in academic achievement. Our findings on the well-being of Canadian adolescents could provide valuable insights for other countries with diverse populations, particularly those with significant immigrant communities.

## Data availability statement

Publicly available datasets were analyzed in this study. This data can be found here: https://www.oecd.org/pisa/data/2018database/.

## Ethics statement

Ethical review and approval was not required for the study on human participants in accordance with the local legislation and institutional requirements. Written informed consent from the patients/participants or patients/participants legal guardian/next of kin was not required to participate in this study in accordance with the national legislation and the institutional requirements.

## Author contributions

YL: Conceptualization, Data curation, Formal analysis, Funding acquisition, Investigation, Methodology, Project administration, Resources, Software, Supervision, Validation, Writing – original draft, Writing – review & editing. NM: Conceptualization, Data curation, Formal analysis, Investigation, Methodology, Resources, Writing – review & editing. MM-B: Conceptualization, Funding acquisition, Investigation, Resources, Validation, Writing – review & editing. SC-B: Conceptualization, Funding acquisition, Investigation, Resources, Validation, Writing – review & editing.

## Footnotes

^1^PISA 2018 documents (e.g., OECD, 2019b) have indicated that 35% of students in Canada had an immigrant background. However, this frequency was adjusted using replicate weights. Demographic statistics provided in our study are based on the raw data.
