# Supplementary material for: Investigating adolescent psychological wellbeing in an educational context using PISA 2018 Canadian data
Source: Front Psychol. 2024 Aug 9;15:1416631. doi: 10.3389/fpsyg.2024.1416631 (PMC11341991; doi:10.3389/fpsyg.2024.1416631)
Supplement: Supplementary file 1 [file Data_Sheet_1.docx]

**Supplemental Materials**

**Table 1.** *Description of Measures and Variables*

| Variable Name | Original Label | Variable Description | Items |
| --- | --- | --- | --- |
| Positive Affect | SWBP | A continuous index based on three 4-point Likert scale items (1=never; 2=rarely; 3=sometimes; 4=always). Positive values in this index mean that the student reported more positive feelings than the average student across OECD countries. | Thinking about yourself and how you normally feel: how often do you feel as described below?   1. Happy 2. Joyful 3. Cheerful |
| Student Self-Efficacy | RESILIENCE | A continuous index based on five 4-point Likert scale items (1=strongly disagree; 2=disagree; 3=agree; 4=strongly agree). Positive values in this index mean that the student reported higher self-efficacy than did the average student across OECD countries. | How much do you agree with the following statements?   1. I usually manage one way or another. 2. I feel proud that I have accomplished things. 3. I feel that I can handle many things at a time. 4. My belief in myself gets me through hard times. 5. When I’m in a difficult situation, I can usually find my way out of it. |
| Fear of Failure | GFOFAIL | A continuous index based on three 4-point Likert scale items (1=strongly disagree; 2=disagree; 3=agree; 4=strongly agree). Positive values in this index mean that the student expressed a greater fear of failure than did the average student across OECD countries. | How much do you agree with the following statements?   1. When I am failing, I worry about what others think of me. 2. When I am failing, I am afraid that I might not have enough talent. 3. When I am failing, this makes me doubt my plans for the future. |
| Mathematics | MATH(PV1-10) | A series of ten continuous plausible values consisting of student achievement scores on the mathematics section of the PISA survey. Raw scores were based on different combinations of the test items for each student. Overall, there were 82 unique mathematics items. The mathematics test items assessed the extent to which students could use their mathematical knowledge and skills to solve various kinds of numerical and spatial problems. | Test items were a mixture of multiple-choice questions and open-ended questions where students provided their own answer. |
| Reading | READ(PV1-10) | A series of ten continuous plausible values consisting of student achievement scores on the literacy section of the PISA survey. Raw scores were based on different combinations of the test items for each student, with multistage adaptive testing adopted as literacy major domain for PISA 2018. Overall, there were 245 unique literacy items and 65 unique fluency sentences. The literacy test items assessed the extent to which students could use their reading skills to understand and interpret the various kinds of written material they are likely to see in everyday life. | Test items were a mixture of multiple-choice questions and open-ended questions where students provided their own answer. |
| Science | SCIE(PV1-10) | A series of ten continuous plausible values consisting of student achievement scores on the science section of the PISA survey. Raw scores were based on different combinations of the test items for each student. Overall, there were 115 unique science items. The science test items assessed the extent to which students could use their scientific knowledge and skills to understand, interpret, and resolve various kinds of scientific challenges. | Test items were a mixture of multiple-choice questions and open-ended questions where students provided their own answer. |
| Sense of Belonging | BELONG | A continuous index based on six 4-point Likert scale items (1=strongly agree; 2=disagree; 3=agree; 4=strongly disagree). Positive values in this index mean that students reported a greater sense of belonging at school than did the average student across OECD countries. | Thinking about your school: to what extent do you agree with the following statements?   1. I feel like an outsider (or left out of things) at school. 2. I make friends easily at school. (R) 3. I feel like I belong at school. (R) 4. I feel awkward and out of place in my school. 5. Other students seem to like me. (R) 6. I feel lonely at school. |
| Exposure to Bullying | BEINGBULLIED | A continuous index based on three 4-point Likert scale items (1=never or almost never; 2=a few times a year; 3= a few times a month; 4=once a week or more). Positive values in this index mean that the student was more exposed to bullying at school than the average student in OECD countries. | During the past 12 months, how often have you had the following experiences in school? (Some experiences can also happen in social media.)   1. Other students left me out of things on purpose. 2. Other students made fun of me. 3. I was threatened by other students. |
| Competitiveness | COMPETE | A continuous index based on three 4-point Likert scale items (1=strongly disagree; 2=disagree; 3=agree; 4=strongly agree). Positive values in this index mean that students reported greater competitiveness than did the average student across OECD countries. | How much do you agree with the following statements about yourself?   1. I enjoy working in situations involving competition with others. 2. It is important for me to perform better than other people on a task. 3. I try harder when I’m in competition with other people. |
| Disciplinary Climate | DISCLIMA | A continuous index based on five 4-point Likert scale items (1=every lesson; 2=most lessons; 3=some lessons; 4=never or hardly ever). Positive values on this variable mean that the student has a better disciplinary climate than the average student across OECD countries. | How often do these things happen in your [test language lessons]?   1. Students don’t listen to what the teacher says. 2. There is noise and disorder. 3. The teacher has to wait a long time for students to quiet down. 4. Students cannot work well. 5. Students don’t start working for a long time after the lesson begins |
| Motivation to Master Tasks | WORKMAST | A continuous index based on three 4-point Likert items (1=strongly disagree; 2=disagree; 3=agree; 4=strongly agree). Positive values in this index mean greater motivation than the average student across OECD countries. | How much do you agree with the following statements about yourself?   1. I find satisfaction in working as hard as I can. 2. Once I start a task, I persist until it is finished. 3. Part of the enjoyment I get from doing things is when I improve on my past performance. |
| Growth Mindset | ST184Q01HA | A binary categorical variable (1=yes growth mindset; 2=no growth mindset) based on 1 4-point Likert scale item (1=strongly disagree; 2=disagree; 3=agree; 4=strongly agree). Students that disagreed with the statement (1 or 2) were coded as having a growth mindset, those that agreed (3 or 4) were coded as not having a growth mindset. The variable was recoded as 0=no growth mindset and 1=yes growth mindset for analyses. | Your intelligence is something about you that you can't change very much. (R) |
| Cognitive Adaptability | COGFLEX | A continuous index based on five 5-point Likert scale items (1=very much like me; 2=mostly like me; 3= somewhat like me; 4=not much like me; 5=not at all like me). All items were reverse coded so that positive values in this index mean that students reported greater cognitive flexibility than did the average student across OECD countries. | How well does each of the following statements below describe you?   1. I can deal with unusual situations. (R) 2. I can change my behaviour to meet the needs of new situations. (R) 3. I can adapt to different situations even when under stress or pressure. (R) 4. When encountering difficult situations with other people, I can think of a way to resolve the situation. (R) 5. I am capable of overcoming my difficulties in interacting with people from other cultures. (R) |
| Perspective Taking | PERSPECT | A continuous index based on four 5-point Likert scale items (1=very much like me; 2=mostly like me; 3= somewhat like me; 4=not much like me; 5=not at all like me). All items were reverse coded so that positive values in this index mean that students reported a greater perspective-taking ability than did the average student across OECD countries. | How well does each of the following statements below describe you?   1. I try to look at everybody's side of a disagreement before I make a decision. (R) 2. I believe that there are two sides to every question and try to look at them both. (R) 3. I sometimes try to understand my friends better by imagining how things look from their perspective. (R) 4. Before criticizing somebody, I try to imagine how I would feel if I were in their place. (R) |
| Respect for People from Other Cultures | RESPECT | A continuous index based on four 5-point Likert scale items (1=very much like me; 2=mostly like me; 3= somewhat like me; 4=not much like me; 5=not at all like me). All items were reverse coded so that positive values in this index mean that students reported greater respect for other cultures than did the average student across OECD countries. | How well does each of the following statements below describe you?   1. I respect people from other cultures as equal human beings. (R) 2. I treat all people with respect regardless of their cultural background. (R) 3. I give space to people from other cultures to express themselves. (R) 4. I respect the values of people from different cultures. (R) |
| Attitudes towards Immigrants | ATTIMM | A continuous index based on four 4-point Likert scale items (1=strongly disagree; 2=disagree; 3=agree; 4=strongly agree). Positive values in this index mean that the student expressed more positive attitudes towards immigrants. | How much do you agree with the following statements about immigrants?   1. Immigrant children should have the same opportunities for education that other children in the country have. 2. Immigrants who live in a country for several years should have the opportunity to vote in elections. 3. Immigrants should have the opportunity to continue their own customs and lifestyle. 4. Immigrants should have all the same rights that everyone else in the country has. |
| Immigration Status | IMMIG | A categorical variable (1=native; 2=second generation; 3= first generation) based on the country of birth of the student and their parents (students were provided a list of countries to choose from). Students are considered native if the student and at least one parent born in the country of assessment. They are considered second generation immigrants if the student was born in the country of assessment, but their parents were born in another country. Students are considered first generation immigrants if both the student and their parents were born outside of the country of assessment. | In what country were you and your parents born?   1. You 2. Mother 3. Father |
| Male | ST004D01T (Gender) | A binary categorical variable (1=female; 2=male) based on the self-reported gender of the student. All other responses were treated as missing. The variable was recoded as 0=female and 1=male for analyses. | Are you female or male? |
| Language at Home | LANGN | A categorical variable based on the language spoken at home by the student. Students were provided a list of languages and asked to select one response. The variable was recoded as 1=English, 2=French, and 3=other for analyses. | What language do you speak at home most of the time? |
| Index of Economic, Social, and Cultural Status | ESCS | A continuous composite score based on three indexes focused on highest parental occupation, parental education, and home possessions, the latter being a proxy for family wealth. Positive values on this variable mean that the student has a greater economic, social, and cultural status than did the average student across OECD countries. | Highest parental occupation: Ordinal index calculated by coding four open-ended questions on the occupational status of the student’s mother and father using four-digit ISCO codes. The higher parental occupation score of either parent or of the only available parent was used for this index. |
|  |  |  | Parental education: Continuous index calculated using an internationally standardized transformation of the highest education level of the student’s parents based on four categorical items. |
|  |  |  | Home possessions: A continuous summary index of all household and possession items. This included three types of questions describing different possessions for a total of 25 items. Higher scores indicate more home possessions. |
| Grade Repetition | REPEAT | A binary variable (0=did not repeat a grade; 1=repeated a grade) based on three 3-point Likert items (1=No, never; 2=Yes, once; 3=Yes, twice or more) focused on grade repetition at various stages of schooling. If students repeated at least one grade, they were coded as 1, if not they were coded as 0. | Have you ever repeated a grade?   1. At [ISCED 1] 2. At [ISCED 2] 3. At [ISCED 3] |
| Public School | SCHLTYPE (School Type) | A binary categorical variable (1=Private Independent; 2= Private Government-dependent; 3=Public) based on two items asking whether the school is public or private according to whether a private entity or a public agency has the ultimate power for decision-making concerning its affairs. The variable was recoded as 0=Private and 1=Public, combining the two private categories for analyses. | 1. Is your school a public or private school? (1=public school; 2= private school) 2. About what percentage of your total funding for a typical school year comes from the following sources? (Percentage, totalling to 100%) 3. Government (includes departments, local, regional, state, and national) 4. Student fees or school charges paid by parents 5. Benefactors, donations, bequests, sponsorships, parent fundraising 6. Other |

*Note.* OECD = Organisation for Economic Cooperation and Development; ISCO = International Standard Classification of Occupations; ISCED = International Standard Classification of Education; (R) = items that were reverse-coded.

**Table 2.** *Results for Positive Affect Based on Four Models (Null, Social and Educational Factors, Immigration, Moderation)*

|  | Model 0 | | Model 1 | | Model 2 | | Model 3 | |
| --- | --- | --- | --- | --- | --- | --- | --- | --- |
|  | Estimate (*SE*) | *t*-test | Estimate (*SE*) | *t*-test | Estimate (*SE*) | *t*-test | Estimate (*SE*) | *t*-test |
| **Fixed Effects** | | | | | | |  |  |
| Intercept | -0.093 (0.015) | -6.274*** | -0.184 (0.041) | -4.476*** | -0.189 (0.041) | -4.581*** | -0.192 (0.040) | -4.746*** |
| ***Control Variables*** |  |  |  |  |  |  |  |  |
| Male |  |  | -0.005 (0.021) | -0.264 | -0.006 (0.021) | -0.288 | -0.005 (0.021) | -0.230 |
| Language (French) ^a^ |  |  | 0.168 (0.025) | 6.630*** | 0.169 (0.025) | 6.664*** | 0.171 (0.025) | 6.772*** |
| Language (other) ^a^ |  |  | 0.063 (0.027) | 2.336* | 0.052 (0.035) | 1.493 | 0.051 (0.035) | 1.458 |
| ESCS |  |  | 0.027 (0.013) | 2.029* | 0.027 (0.013) | 2.040* | 0.027 (0.013) | 2.073* |
| Grade Repetition |  |  | 0.033 (0.044) | 0.748 | 0.032 (0.044) | 0.732 | 0.036 (0.045) | 0.804 |
| Public School |  |  | 0.107 (0.034) | 3.157** | 0.109 (0.034) | 3.209*** | 0.113 (0.034) | 3.349** |
| ***Primary Predictors*** |  |  |  |  |  |  |  |  |
| Sense of Belonging |  |  | 0.327 (0.012) | 26.696*** | 0.327 (0.012) | 26.699*** | 0.327 (0.012) | 26.586*** |
| Exposure to Bullying |  |  | -0.094 (0.009) | -9.866*** | -0.094 (0.009) | -9.876*** | -0.093 (0.009) | -9.856*** |
| Competitiveness |  |  | 0.041 (0.011) | 3.695*** | 0.041 (0.011) | 3.660*** | 0.040 (0.011) | 3.657*** |
| Disciplinary Climate |  |  | 0.020 (0.010) | 1.994* | 0.019 (0.010) | 1.980* | 0.019 (0.010) | 1.985* |
| Motivation |  |  | 0.135 (0.011) | 11.991*** | 0.135 (0.011) | 11.991*** | 0.133 (0.011) | 11.734*** |
| Growth Mindset |  |  | 0.012 (0.025) | 0.462 | 0.011 (0.026) | 0.426 | 0.011 (0.025) | 0.440 |
| Cognitive Adaptability |  |  | 0.081 (0.013) | 6.326*** | 0.081 (0.013) | 6.318*** | 0.082 (0.013) | 6.387*** |
| Perspective Taking |  |  | -0.016 (0.013) | -1.252 | -0.016 (0.013) | -1.260 | -0.017 (0.013) | -1.292 |
| Respect |  |  | 0.032 (0.015) | 2.066* | 0.031 (0.015) | 2.057* | 0.026 (0.017) | 1.527 |
| Img1Gen |  |  |  |  | 0.019 (0.036) | 0.512 | -0.016 (0.043) | -0.363 |
| Img2Gen |  |  |  |  | 0.016 (0.029) | 0.572 | -0.004 (0.035) | -0.113 |
| AttdImg |  |  |  |  |  |  | -0.004 (0.013) | -0.310 |
| Img1Gen * AttdImg |  |  |  |  |  |  | 0.071 (0.034) | 2.109* |
| Img2Gen * AttdImg |  |  |  |  |  |  | 0.042 (0.030) | 1.417 |
| **Random Effects** |  |  |  |  |  |  |  |  |
| Residual Variance | 1.054 |  | 0.837 |  | 0.837 |  | 0.837 |  |
| School Variance | 0.027 |  | 0.005 |  | 0.005 |  | 0.005 |  |
| **R Square** |  |  |  |  |  |  |  |  |
| Within-Level |  |  | 0.222 |  | 0.222 |  | 0.223 |  |
| Between-Level |  |  | 0.225 |  | 0.233 |  | 0.246 |  |

*Note*. ESCS = Index of economic, social, and cultural status; Motivation = Motivation to master tasks; Respect = Respect for people from other cultures; Img1Gen = 1^st^ generation immigrants; Img2Gen= 2^nd^ generation immigrants; AttdImg= Attitudes towards immigrants; ^a^ Reference category is English; **p* ≤ 0.05, ***p* < .01, ****p* < .001.

**Table 3.** *Results for Self-Efficacy Based on Four Models (Null, Social and Educational Factors, Immigration, Moderation)*

|  | Model 0 | | Model 1 | | Model 2 | | Model 3 | |
| --- | --- | --- | --- | --- | --- | --- | --- | --- |
|  | Estimate (*SE*) | *t*-test | Estimate (*SE*) | *t*-test | Estimate (*SE*) | *t*-test | Estimate (*SE*) | *t*-test |
| **Fixed Effects** | | | | | | |  |  |
| Intercept | 0.112 (0.012) | 9.393*** | -0.037 (0.039) | -0.930 | -0.048 (0.039) | -1.228 | -0.054 (0.038) | -1.413 |
| ***Control Variables*** |  |  |  |  |  |  |  |  |
| Male |  |  | 0.093 (0.017) | 5.366*** | 0.092 (0.017) | 5.305*** | 0.095 (0.017) | 5.533*** |
| Language (French) ^a^ |  |  | 0.079 (0.026) | 3.079** | 0.082 (0.025) | 3.224** | 0.084 (0.025) | 3.297** |
| Language (other) ^a^ |  |  | 0.082 (0.024) | 3.470** | 0.056 (0.028) | 1.979* | 0.055 (0.028) | 1.930 |
| ESCS |  |  | 0.034 (0.010) | 3.313** | 0.034 (0.010) | 3.355** | 0.034 (0.010) | 3.421** |
| Grade Repetition |  |  | 0.015 (0.053) | 0.282 | 0.014 (0.053) | 0.265 | 0.018 (0.053) | 0.346 |
| Public School |  |  | -0.009 (0.036) | -0.247 | -0.004 (0.035) | -0.117 | 0.001 (0.035) | 0.015 |
| ***Primary Predictors*** |  |  |  |  |  |  |  |  |
| Sense of Belonging |  |  | 0.160 (0.010) | 15.724*** | 0.160 (0.010) | 15.707*** | 0.159 (0.010) | 15.584*** |
| Exposure to Bullying |  |  | -0.013 (0.009) | -1.406 | -0.013 (0.009) | -1.414 | -0.012 (0.009) | -1.324 |
| Competitiveness |  |  | 0.098 (0.011) | 9.221*** | 0.098 (0.011) | 9.171*** | 0.097 (0.011) | 9.188*** |
| Disciplinary Climate |  |  | 0.020 (0.009) | 2.128* | 0.019 (0.009) | 2.037* | 0.019 (0.009) | 2.026* |
| Motivation |  |  | 0.311 (0.012) | 26.448*** | 0.311 (0.012) | 26.470*** | 0.307 (0.012) | 25.982*** |
| Growth Mindset |  |  | 0.032 (0.019) | 1.654 | 0.029 (0.019) | 1.539 | 0.029 (0.019) | 1.533 |
| Cognitive Adaptability |  |  | 0.293 (0.013) | 22.627*** | 0.293 (0.013) | 22.648*** | 0.294 (0.013) | 22.813*** |
| Perspective Taking |  |  | -0.005 (0.010) | -0.451 | -0.005 (0.010) | -0.497 | -0.006 (0.010) | -0.567 |
| Respect |  |  | -0.005 (0.012) | -0.418 | -0.005 (0.012) | -0.452 | -0.016 (0.013) | -1.253 |
| Img1Gen |  |  |  |  | 0.040 (0.031) | 1.281 | 0.003 (0.038) | 0.091 |
| Img2Gen |  |  |  |  | 0.040 (0.022) | 1.788 | 0.011 (0.024) | 0.450 |
| AttdImg |  |  |  |  |  |  | 0.004 (0.013) | 0.341 |
| Img1Gen * AttdImg |  |  |  |  |  |  | 0.072 (0.031) | 2.346* |
| Img2Gen * AttdImg |  |  |  |  |  |  | 0.056 (0.023) | 2.414* |
| **Random Effects** |  |  |  |  |  |  |  |  |
| Residual Variance | 1.004 |  | 0.633 |  | 0.633 |  | 0.632 |  |
| School Variance | 0.015 |  | 0.005 |  | 0.005 |  | 0.005 |  |
| **R Square** |  |  |  |  |  |  |  |  |
| Within-Level |  |  | 0.376 |  | 0.376 |  | 0.377 |  |
| Between-Level |  |  | 0.002 |  | 0.001 |  | 0.001 |  |

*Note*. Same notes as Table 2 above.

**Table 4.** *Results for Fear of Failure Based on Four Models (Null, Social and Educational Factors, Immigration, Moderation)*

|  | Model 0 | | Model 1 | | Model 2 | | Model 3 | |
| --- | --- | --- | --- | --- | --- | --- | --- | --- |
|  | Estimate (*SE*) | *t*-test | Estimate (*SE*) | *t*-test | Estimate (*SE*) | *t*-test | Estimate (*SE*) | *t*-test |
| **Fixed Effects** | | | | | | |  |  |
| Intercept | 0.218 (0.012) | 17.561*** | 0.476 (0.040) | 11.963*** | 0.462 (0.040) | 11.529*** | 0.442 (0.040) | 11.065*** |
| ***Control Variables*** |  |  |  |  |  |  |  |  |
| Male |  |  | -0.471 (0.025) | -19.057*** | -0.473 (0.025) | -19.080*** | -0.462 (0.025) | -18.571*** |
| Language (French) ^a^ |  |  | -0.111 (0.027) | -4.183*** | -0.107 (0.027) | -4.006*** | -0.108 (0.027) | -4.030*** |
| Language (other) ^a^ |  |  | -0.035 (0.028) | -1.251 | -0.045 (0.031) | -1.421 | -0.047 (0.031) | -1.508 |
| ESCS |  |  | 0.066 (0.013) | 5.161*** | 0.067 (0.013) | 5.238*** | 0.064 (0.013) | 5.012*** |
| Grade Repetition |  |  | -0.041 (0.052) | -0.796 | -0.038 (0.052) | -0.733 | -0.032 (0.052) | -0.612 |
| Public School |  |  | 0.042 (0.034) | 1.223 | 0.047 (0.034) | 1.388 | 0.049 (0.033) | 1.461 |
| ***Primary Predictors*** |  |  |  |  |  |  |  |  |
| Sense of Belonging |  |  | -0.160 (0.014) | -11.461*** | -0.160 (0.014) | -11.495*** | -0.162 (0.014) | -11.808*** |
| Exposure to Bullying |  |  | 0.102 (0.013) | 8.156*** | 0.102 (0.013) | 8.147*** | 0.103 (0.013) | 8.218*** |
| Competitiveness |  |  | 0.104 (0.011) | 9.612*** | 0.104 (0.011) | 9.550*** | 0.103 (0.011) | 9.582*** |
| Disciplinary Climate |  |  | 0.009 (0.012) | 0.742 | 0.009 (0.012) | 0.738 | 0.008 (0.012) | 0.696 |
| Motivation |  |  | 0.088 (0.013) | 6.970*** | 0.088 (0.013) | 6.950*** | 0.081 (0.013) | 6.360*** |
| Growth Mindset |  |  | -0.191 (0.020) | -9.334*** | -0.194 (0.021) | -9.433*** | -0.195 (0.020) | -9.567*** |
| Cognitive Adaptability |  |  | -0.134 (0.013) | -10.423*** | -0.133 (0.013) | -10.376*** | -0.130 (0.013) | -10.192*** |
| Perspective Taking |  |  | 0.071 (0.013) | 5.598*** | 0.070 (0.013) | 5.489*** | 0.068 (0.013) | 5.301*** |
| Respect |  |  | 0.116 (0.014) | 8.066*** | 0.115 (0.014) | 7.998*** | 0.086 (0.015) | 5.888*** |
| Img1Gen |  |  |  |  | -0.002 (0.028) | -0.088 | -0.024 (0.030) | -0.776 |
| Img2Gen |  |  |  |  | 0.085 (0.033) | 2.552* | 0.078 (0.038) | 2.029* |
| AttdImg |  |  |  |  |  |  | 0.061 (0.014) | 4.265*** |
| Img1Gen * AttdImg |  |  |  |  |  |  | 0.022 (0.027) | 0.840 |
| Img2Gen * AttdImg |  |  |  |  |  |  | -0.005 (0.037) | -0.121 |
| **Random Effects** |  |  |  |  |  |  |  |  |
| Residual Variance | 1.083 |  | 0.922 |  | 0.922 |  | 0.918 |  |
| School Variance | 0.015 |  | 0.004 |  | 0.004 |  | 0.004 |  |
| **R Square** |  |  |  |  |  |  |  |  |
| Within-Level |  |  | 0.156 |  | 0.157 |  | 0.160 |  |
| Between-Level |  |  | 0.053 |  | 0.075 |  | 0.076 |  |

*Note.* Same notes as Table 2 above.

**Table 5.** *Results for Mathematics Achievement Based on Three Models (Null, Control Variables Only, Psychological Well-being)*

|  | Model 0 | | Model 1 | | Model 2 | |
| --- | --- | --- | --- | --- | --- | --- |
|  | Estimate (*SE*) | *t*-test | Estimate (*SE*) | *t*-test | Estimate (*SE*) | *t*-test |
| **Fixed Effects** | | | | | | |
| Intercept | 501.635 (3.339) | 150.252*** | 493.245 (9.686) | 50.926*** | 491.139 (9.759) | 50.324*** |
| ***Control Variables*** |  |  |  |  |  |  |
| Male |  |  | 17.825 (2.265) | 7.871*** | 18.548 (2.353) | 7.883*** |
| Language (French) ^a^ |  |  | 18.990 (5.470) | 3.471** | 19.491 (5.479) | 3.557*** |
| Language (Other) ^a^ |  |  | 7.893 (3.454) | 2.285* | 8.089 (3.427) | 2.360* |
| ESCS |  |  | 19.213 (1.216) | 15.795*** | 19.044 (1.241) | 15.348*** |
| Grade Repetition |  |  | -50.497 (5.394) | -9.362*** | -50.316 (5.382) | -9.350*** |
| Public School |  |  | -25.087 (8.723) | -2.876** | -24.453 (8.712) | -2.807** |
| Sense of Belonging |  |  | -5.640 (1.590) | -3.548*** | -3.957 (1.639) | -2.415* |
| Exposure to Bullying |  |  | -6.202 (1.197) | -5.183*** | -6.991 (1.180) | -5.924*** |
| Competitiveness |  |  | 3.692 (1.290) | 2.863** | 3.195 (1.295) | 2.467* |
| Disciplinary Climate |  |  | 4.599 (1.127) | 4.080*** | 4.638 (1.135) | 4.086*** |
| Motivation |  |  | 5.506 (1.628) | 3.382** | 4.685 (1.628) | 2.877** |
| Growth Mindset |  |  | 17.839 (2.793) | 6.386*** | 18.265 (2.811) | 6.498*** |
| Cognitive Adaptability |  |  | 1.337 (1.264) | 1.058 | 0.800 (1.314) | 0.609 |
| Perspective Taking |  |  | -1.356 (1.810) | -0.749 | -1.591 (1.833) | -0.868 |
| Respect |  |  | 9.602 (1.379) | 6.961*** | 9.582 (1.356) | 7.068*** |
| Img1Gen |  |  | -14.394 (4.211) | -3.418** | -14.377 (4.195) | -3.427** |
| Img2Gen |  |  | -9.563 (4.372) | -2.187* | -9.719 (4.382) | -2.218* |
| AttdImg |  |  | 9.639 (1.434) | 6.722*** | 9.447 (1.431) | 6.604*** |
| ***Primary Predictors*** |  |  |  |  |  |  |
| Positive Affect |  |  |  |  | -6.168 (1.422) | -4.337*** |
| Self-Efficacy |  |  |  |  | 4.639 (1.778) | 2.609** |
| Fear of Failure |  |  |  |  | 2.637 (0.947) | 2.785** |
| **Random Effects** |  |  |  |  |  |  |
| Residual Variance | 6928.814 |  | 5973.898 |  | 5929.686 |  |
| School Variance | 1648.358 |  | 891.390 |  | 891.884 |  |
| **R Square** |  |  |  |  |  |  |
| Within-Level |  |  | 0.168 |  | 0.175 |  |
| Between-Level |  |  | 0.089 |  | 0.085 |  |

*Note*. Same notes as Table 2 above.

**Table 6.** *Results for Reading Achievement Based on Three Models (Null, Control Variables Only, Psychological Well-being)*

|  | Model 0 | | Model 1 | | Model 2 | |
| --- | --- | --- | --- | --- | --- | --- |
|  | Estimate (*SE*) | *t*-test | Estimate (*SE*) | *t*-test | Estimate (*SE*) | *t*-test |
| **Fixed Effects** | | | | | | |
| Intercept | 507.517 (2.786) | 182.154*** | 506.408 (9.560) | 52.971*** | 502.358 (9.361) | 53.665*** |
| ***Control Variables*** |  |  |  |  |  |  |
| Male |  |  | -10.190 (1.963) | -5.190*** | -7.133 (1.957) | -3.644*** |
| Language (French) ^a^ |  |  | 3.008 (4.339) | 0.693 | 3.921 (4.306) | 0.911 |
| Language (Other) ^a^ |  |  | -12.295 (3.370) | -3.649*** | -11.724 (3.356) | -3.494*** |
| ESCS |  |  | 16.731 (1.230) | 13.599*** | 16.456 (1.224) | 13.443*** |
| Grade Repetition |  |  | -46.548 (4.531) | -10.274*** | -46.221 (4.570) | -10.113*** |
| Public School |  |  | -17.414 (9.393) | -1.854 | -17.106 (9.279) | -1.844 |
| Sense of Belonging |  |  | -6.757 (1.178) | -5.738*** | -3.755 (1.364) | -2.753** |
| Exposure to Bullying |  |  | -6.088 (0.993) | -6.133*** | -7.402 (1.013) | -7.310*** |
| Competitiveness |  |  | 2.973 (1.006) | 2.955** | 2.330 (1.028) | 2.268* |
| Disciplinary Climate |  |  | 5.419 (1.069) | 5.070*** | 5.488 (1.087) | 5.050*** |
| Motivation |  |  | 6.704 (1.094) | 6.127*** | 6.441 (1.157) | 5.568*** |
| Growth Mindset |  |  | 22.870 (2.174) | 10.521*** | 24.227 (2.199) | 11.016*** |
| Cognitive Adaptability |  |  | -0.557 (1.210) | -0.460 | 0.363 (1.227) | 0.295 |
| Perspective Taking |  |  | -0.784 (1.118) | -0.701 | -1.339 (1.140) | -1.175 |
| Respect |  |  | 16.034 (1.341) | 11.957*** | 15.618 (1.347) | 11.596*** |
| Img1Gen |  |  | -19.455 (3.184) | -6.110*** | -19.313 (3.154) | -6.123*** |
| Img2Gen |  |  | -6.079 (3.106) | -1.957* | -6.440 (3.102) | -2.076* |
| AttdImg |  |  | 14.782 (1.052) | 14.046*** | 14.373 (1.045) | 13.756*** |
| ***Primary Predictors*** |  |  |  |  |  |  |
| Positive Affect |  |  |  |  | -6.557 (1.166) | -5.622*** |
| Self-Efficacy |  |  |  |  | 1.766 (1.324) | 1.334 |
| Fear of Failure |  |  |  |  | 7.106 (0.986) | 7.204*** |
| **Random Effects** |  |  |  |  |  |  |
| Residual Variance | 8650.885 |  | 7077.910 |  | 6999.819 |  |
| School Variance | 1646.014 |  | 870.254 |  | 845.044 |  |
| **R Square** |  |  |  |  |  |  |
| Within-Level |  |  | 0.211 |  | 0.221 |  |
| Between-Level |  |  | 0.046 |  | 0.046 |  |

*Note*. Same notes as Table 2 above.

**Table 7.** *Results for Science Achievement Based on Three Models (Null, Control Variables Only, Psychological Well-being)*

|  | Model 0 | | Model 1 | | Model 2 | |
| --- | --- | --- | --- | --- | --- | --- |
|  | Estimate (*SE*) | *t*-test | Estimate (*SE*) | *t*-test | Estimate (*SE*) | *t*-test |
| **Fixed Effects** | | | | | | |
| Intercept | 506.542 (3.135) | 161.555*** | 495.108 (10.435) | 47.446*** | 491.489 (10.219) | 48.098*** |
| ***Control Variables*** |  |  |  |  |  |  |
| Male |  |  | 10.946 (2.774) | 3.946*** | 13.477 (2.751) | 4.900*** |
| Language (French) ^a^ |  |  | 5.444 (4.683) | 1.163 | 6.329 (4.701) | 1.346 |
| Language (Other) ^a^ |  |  | -10.433 (3.868) | -2.697** | -9.896 (3.940) | -2.512* |
| ESCS |  |  | 15.967 (1.298) | 12.298*** | 15.775 (1.306) | 12.075*** |
| Grade Repetition |  |  | -45.057 (4.977) | -9.053*** | -44.754 (4.991) | -8.967*** |
| Public School |  |  | -11.834 (10.366) | -1.142 | -11.420 (10.205) | -1.119 |
| Sense of Belonging |  |  | -6.197 (1.218) | -5.086*** | -3.246 (1.491) | -2.177* |
| Exposure to Bullying |  |  | -5.142 (1.302) | -3.949*** | -6.369 (1.372) | -4.643*** |
| Competitiveness |  |  | 5.997 (1.193) | 5.028*** | 5.508 (1.241) | 4.440*** |
| Disciplinary Climate |  |  | 4.538 (1.182) | 3.841*** | 4.630 (1.189) | 3.895*** |
| Motivation |  |  | 4.623 (1.060) | 4.359*** | 4.566 (1.105) | 4.134*** |
| Growth Mindset |  |  | 15.952 (2.445) | 6.524*** | 17.094 (2.374) | 7.201*** |
| Cognitive Adaptability |  |  | 1.408 (1.313) | 1.073 | 2.261 (1.360) | 1.662 |
| Perspective Taking |  |  | -0.422 (1.314) | -0.321 | -0.905 (1.316) | -0.688 |
| Respect |  |  | 11.568 (1.550) | 7.462*** | 11.257 (1.525) | 7.379*** |
| Img1Gen |  |  | -16.838 (3.798) | -4.433*** | -16.695 (3.763) | -4.437*** |
| Img2Gen |  |  | -9.025 (3.537) | -2.552* | -9.305 (3.542) | -2.627** |
| AttdImg |  |  | 13.397 (1.511) | 8.868*** | 13.076 (1.525) | 8.575*** |
| ***Primary Predictors*** |  |  |  |  |  |  |
| Positive Affect |  |  |  |  | -6.904 (1.441) | -4.791*** |
| Self-Efficacy |  |  |  |  | 1.566 (1.810) | 0.865 |
| Fear of Failure |  |  |  |  | 5.917 (1.149) | 5.149*** |
| **Random Effects** |  |  |  |  |  |  |
| Residual Variance | 7838.384 |  | 6775.378 |  | 6705.559 |  |
| School Variance | 1574.620 |  | 938.135 |  | 914.334 |  |
| **R Square** |  |  |  |  |  |  |
| Within-Level |  |  | 0.163 |  | 0.172 |  |
| Between-Level |  |  | 0.021 |  | 0.020 |  |

*Note*. Same notes as Table 2 above.
